# Modeling the positive testing rate of COVID-19 in South Africa using a semi-parametric smoother for binomial data

**DOI:** 10.3389/fpubh.2023.979230

**Published:** 2023-02-22

**Authors:** Olajumoke Evangelina Owokotomo, Samuel Manda, Jürgen Cleasen, Adetayo Kasim, Rudradev Sengupta, Rahul Shome, Soumya Subhra Paria, Tarylee Reddy, Ziv Shkedy

**Affiliations:** ^1^Center for Statistics, Data Science Institute, I-BioStat, Hasselt University, Hasselt, Belgium; ^2^Department of Statistics, University of Pretoria, Pretoria, South Africa; ^3^Department of Anthropology, Durham Research Methods Centre, Durham University, Durham, United Kingdom; ^4^The Janssen Pharmaceutical, Companies of Johnson & Johnson, Beerse, Belgium; ^5^Department of Computer Science, Rice University, Houston, TX, United States; ^6^School of Mathematics and Statistics, The Open University, Milton Keynes, United Kingdom; ^7^Biostatistics Research Unit, South African Medical Research Council, Capetown, South Africa

**Keywords:** COVID-19, South Africa, positive testing rate, semi-parametric smoothing model, transmission rates

## Abstract

Identification and isolation of COVID-19 infected persons plays a significant role in the control of COVID-19 pandemic. A country's COVID-19 positive testing rate is useful in understanding and monitoring the disease transmission and spread for the planning of intervention policy. Using publicly available data collected between March 5th, 2020 and May 31st, 2021, we proposed to estimate both the positive testing rate and its daily rate of change in South Africa with a flexible semi-parametric smoothing model for discrete data. There was a gradual increase in the positive testing rate up to a first peak rate in July, 2020, then a decrease before another peak around mid-December 2020 to mid-January 2021. The proposed semi-parametric smoothing model provides a data driven estimates for both the positive testing rate and its change. We provide an online R dashboard that can be used to estimate the positive rate in any country of interest based on publicly available data. We believe this is a useful tool for both researchers and policymakers for planning intervention and understanding the COVID-19 spread.

## 1. Introduction

Coronaviruses are a large family of viruses which may cause respiratory infections ranging from the common cold to more severe diseases such as Middle East respiratory syndrome (MERS) and Severe acute respiratory syndrome (SARS). The ongoing outbreak of the novel coronavirus SARS-CoV-2 was first reported in December 2019, in Wuhan, China (1, 2). The virus has rapidly spread with a total of 243,260,214 confirmed cases and 4,941,039 deaths as of October 25th, 2021 (2). South Africa was one of the first African countries to initiate containment measures against COVID-19. The country was experiencing higher numbers of COVID-19 cases compared to most countries in Sub-Saharan Africa. The first reported COVID-19 cases in South Africa were related to nine adults who returned from Italy, where the infection rate was uncontrolled (3). After showing symptoms of flu, the 9 subjects were confirmed as COVID-19 positive through the reverse transcription-polymerase chain reaction test on March 5th, 2020. As COVID-19 cases increased in South Africa and no availability of approved vaccines, the authorities and health system in the country imposed compulsory measures in addition to the recommendations from world health organisation (WHO) and the strategies from Africa centers for disease control and prevention [Africa CDC, (4)]. Foreigners from high-risk countries were banned from traveling into the country and restrictions were placed on non-essential domestic and international outgoing flights. South African citizens returning from high-risk countries had to self-quarantined on arrival, individuals who had contact with infected patients were traced and asked to self-isolate. The majority of the entry ports in the country were shut down as well. In addition, all schools were closed and gatherings of more than 100 people became impermissible. The after-effect of these cases propelled the South African government to declare a national state of disaster on March 15th, 2020 followed by a 21-day lockdown period which commenced on March 27th, 2020 (5–7). Awareness campaigns were intensified to fight anxiety, depression, stigmatization, myths and misinformation about COVID-19. Media platforms such as television, radio, social media, short messaging services (SMS), leaflets, banners, and also road campaigns were used to create awareness. Hand-washing techniques and preventive measures such as mask-wearing, sanitizing, and social distancing were also included in the awareness campaigns (4).

The lockdown was eased in June 2020, the entry ports were opened and people began to return to work gradually. As expected, the rate of infections and deaths began to increase again leading to the anticipation of a second wave (8). To contain the spread of the virus during the second wave, South Africa maintained interventions such as travel restrictions, public gatherings with a limited number of people in attendance, social distancing, hand sanitizing, and mask-wearing (9). Temperature screening was carried out at entry ports. In addition, laboratory testing facilities, clinical diagnosis, quarantine facilities, and reconstruction of some selected hospitals as COVID-19 isolation centers were established in each province of the country. The peak of the second wave was observed on January 8th, 2021 with 21,980 COVID-19 cases diagnosed. Contact tracking and data collection were carried out for people who tested positive to the virus; the data collected included symptoms, travel details, exposure to anyone infected, exposure to healthcare facilities, and contact details of the person (4). Toward the end of 2020, a new variant of the COVID-19 virus was identified. The spread of the new variant was more rapid than the original variant and this increased the pressure on the health system. In response to the new variant, South Africa closed the borders for general entry and departure from January 11th, 2021 until Febuary 15th, 2021 (9). On September, 2021, South Africa has incurred about 20 million doses of vaccine from different manufacturers, with the aim of vaccinating at least 67% of its population by the end of the year 2021 (9, 10).

**Figure 1:**
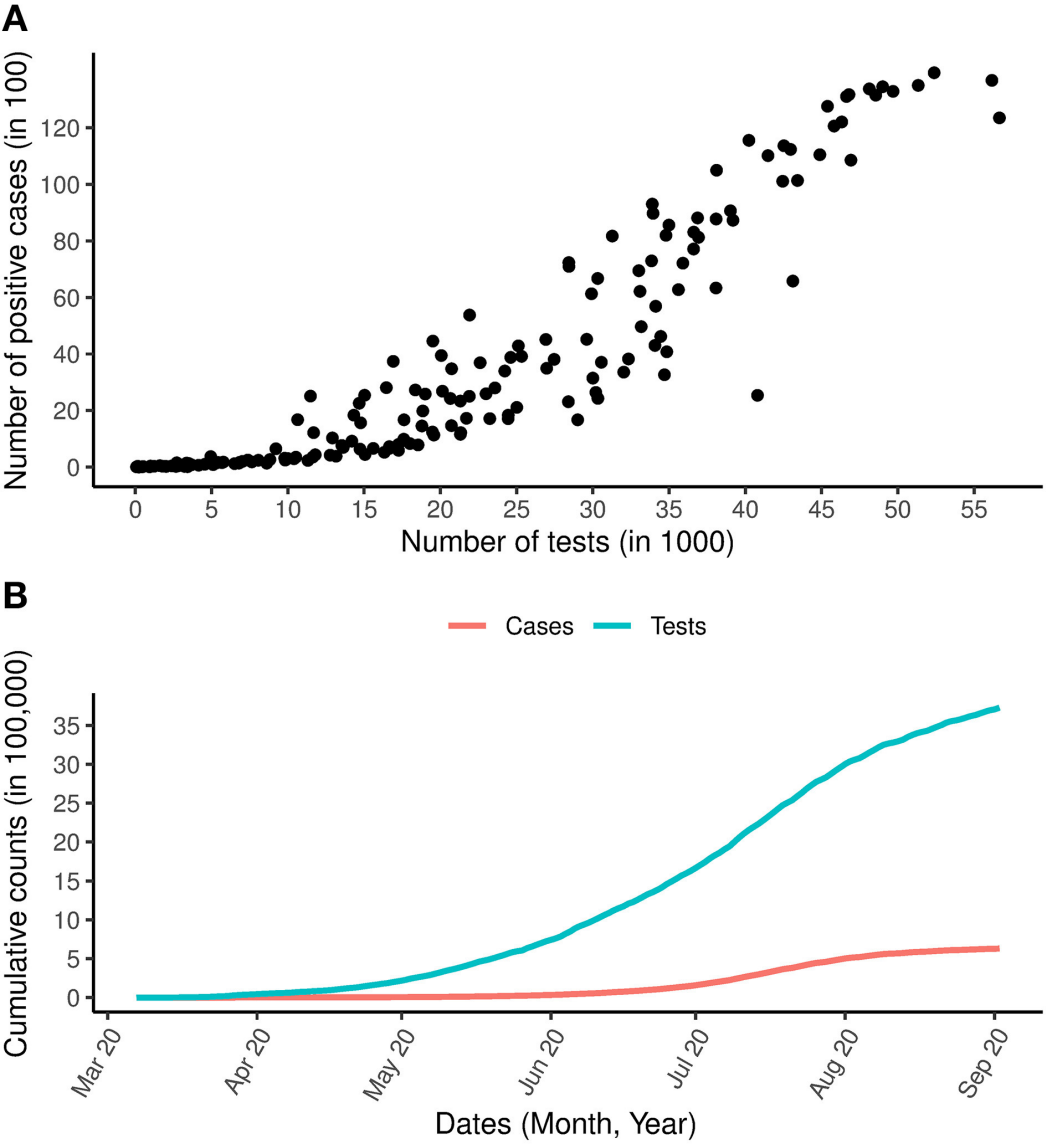
COVID-19 cases and tests over time. **(A)** Is the relationship between the daily number of COVID-19 tests and the daily number of COVID-19 positive cases. **(B)** Is the total number of COVID-19 cases and total number of COVID-19 tests carried out between the period March 7th, 2020 and September 2nd, 2020.

Modeling the number of COVID-19 cases, and in particular, producing a reliable short and long term predictions of the number of COVID-19 cases are critical tools for policy makers to design interventions in order to control the spread of the disease. Recently, Reddy et al. (6) applied a robust model-based approach, that does not require making assumptions about the transmission process to model the number of COVID-19 cases and they were able to provide accurate short term prediction for 5–10 days using the COVID-19 data from South Africa. These non-linear epidemiological models have previously been applied to model other disease outbreaks such as Ebola (11), Dengue (12), Zika virus (13) and, more recently, the COVID-19 pandemic (14–16). Specifically, Roosa et al. (14) fitted the generalized logistic model, Richards's model and a sub-epidemic model to the cumulative COVID-19 cases in the Hubei province of China and produced a short-term forecast of 5, 10, and 15 days ahead. In a recent analysis by Shen (15), a similar approach was used to estimate the key epidemic parameters for all 11 provinces in China as well as 9 selected countries. All the models discussed in the literature above made use of the daily or cumulative number of cases to fit the models, estimate the parameters of interest and to provide a short-term prediction of the number of COVID-19 cases. In the context of COVID-19, using the number of cases alone might not be sufficient because, as seen in Figure 1A, for the South Africa case study (and in many other countries), there is a strong positive correlation between the number of cases and the number of tests performed (Spearmans correlation = 0.96, *p* < 0.0005). The cumulative number of tests and cases shown in Figure 1B reveals a similar trend for cases and tests over time. Therefore, the positive testing rate may provide more reliable insights on the epidemic evolution and effects of interventions (non-pharmaceutical interventions and vaccinations) as the positive testing rate effectively adjusts the number of cases diagnosed for the number of tests performed (6).

In addition to the short-term predictive models discussed above, a large amount of recent research related to COVID-19 modeling has been published using different forms of compartmental models. In particular, studies of Iyiola et al. (17) and Owusu-Mensah et al. (18) proposed a generalized compartmental model with various disease-specific parameters. The models presented in Iyiola et al. (17), Owusu-Mensah et al. (18), and Iyiola et al. (19) were developed to provide a better insight into controlling the spread of the disease. The possibility of a third and fourth wave in the spike of COVID-19 was predicted using these models. Social distancing, the use of masks, and aggressive testing were highly recommended based on the results reported in Iyiola et al. (17) and Owusu-Mensah et al. (18), and tracing was seen to be important in reducing the number of infected individuals in public. Furthermore, deep learning algorithms a data analysis approach have been extensively used in literature as a non-surgical technique in reducing the burden of COVID-19. In addition, it provides the best possible means for diagnosis and prognosis (20–22). Muhammad et al. (23) proposed various supervised learning algorithms to classify individuals with positive and negative COVID-19 cases, Muhammad et al. (24) developed data mining models for predicting COVID-19 infected patients recovery using epidemiological dataset of COVID-19 patients in South Korea. Their model predicted an interval with minimum and maximum number of days for COVID-19 patients to recover from the virus and age group of patients who are likely to recover. In both research, models developed with decision tree mining algorithm out-performs other algorithms with highest accuracy and prediction power. We follow the same goal of understanding the spread of COVID-19 using a different modeling approach, as we do not aim to predict future cases but rather to provide an exploratory tool to model the positive testing rate.

Positive testing rate, which refers to the number of positive COVID-19 tests divided by the number of COVID-19 tests in a prescribed period, has been seen as an important statistic in understanding the transmission of COVID-19 (25). Due to the correlation between the number of COVID-19 cases and the number of COVID-19 tests conducted, no country would be able to know the actual total number of people infected with COVID-19 but only the infection status of those who have been tested. Therefore, in countries with a high positivity rate, the number of confirmed COVID-19 cases is more likely to represent only a small proportion of the true number of cases. However, if the probability of positive tests increases then it suggests the virus is spreading faster than the growth seen in confirmed cases. The positivity rate is of great importance, and it is used to (1) guide policy makers on COVID-19 interventions and decision-making, (2) for surveillance purposes and (3) decide whether to relax or impose restrictions aimed at slowing down the spread of COVID-19 transmission. This was evidenced on May 12th, 2020 when the WHO advised governments that before relaxing intervention measures, the positive testing rate should remain at 5% or lower for at least 14 days (26). Recently, the center for disease control and prevention issued guidelines on the calculation of the positive testing rate as an important measure for public health surveillance (27). The relationship between demographic factors and the positive testing rate in specimens from a particular hospital in Wuhan, China was reported by Liu et al. (28). Other authors, through examination of the daily COVID-19 incidence and testing, showed that changes in testing rates could mask the epidemic's growth rate, which has public health implications (29). In addition, authors are now attempting to estimate the state-level COVID-19 prevalence in the United States using COVID-19 positive testing rate (30). To our knowledge, there have been limited efforts to model directly the COVID-19 positive testing rate and the rate of change over time.

To provide a more accurate perspective on the disease burden, we propose a modeling approach that focuses on COVID-19 positive testing rate, i.e., the probability of positive cases per tests conducted and the rate of change in this rate over time. In this paper we proposed to model the daily number of COVID-19 cases among the number of COVID-19 tests carried out using a semi-parametric model in which the rate of change of the positive testing rate is estimated using a smooth function of time. In particular, we apply scatter plot smoothing techniques for binomial data using generalized additive models [GAM, (31)] in order to obtain estimates for both the positive testing rate and its rate of change over time. The advantage of the proposed model is that it is applied directly to the observed data and therefore can accommodate changes in the positive rate caused by implementation of different interventions activities such as lockdown, testing strategy, and vaccination policy. Hence, the proposed model can be used for both the evaluation of a specific intervention (or combination of interventions) and understanding the trend over time in the country. For the latter, we proposed the rate of change of the positive testing rate, i.e., the first derivative of the positive testing rate with respect to time. We illustrate the proposed method using the COVID-19 dataset from South Africa. The models and methods discussed in this paper were also applied to four additional countries, Poland, UK, Ethiopia and India, for which different testing strategies and vaccination programmes were implemented (and different vaccination coverage were achieved). The results for these countries are presented in the Supplementary material for the paper. In addition, an online R dashboard (32) was developed to estimate and visualize the positive testing rate and the rate of change using a publicly available dataset (33) using the methodology discussed in this paper.

The remainder of this paper is organized as follows. We begin by describing the testing policy in South Africa from which the data used for the analysis presented in this paper was obtained. The modeling approach, the model formulation for the positive testing rate and the methodology to construct simultaneous confidence bands were then explained followed by the results obtained for South Africa.

## 2. Materials and methods

### 2.1. Data

#### 2.1.1. Daily number of tests and confirmed cases

##### 2.1.1.1. First-wave in South Africa: March, 7th 2020–September, 2nd 2020

**Figure 2:**
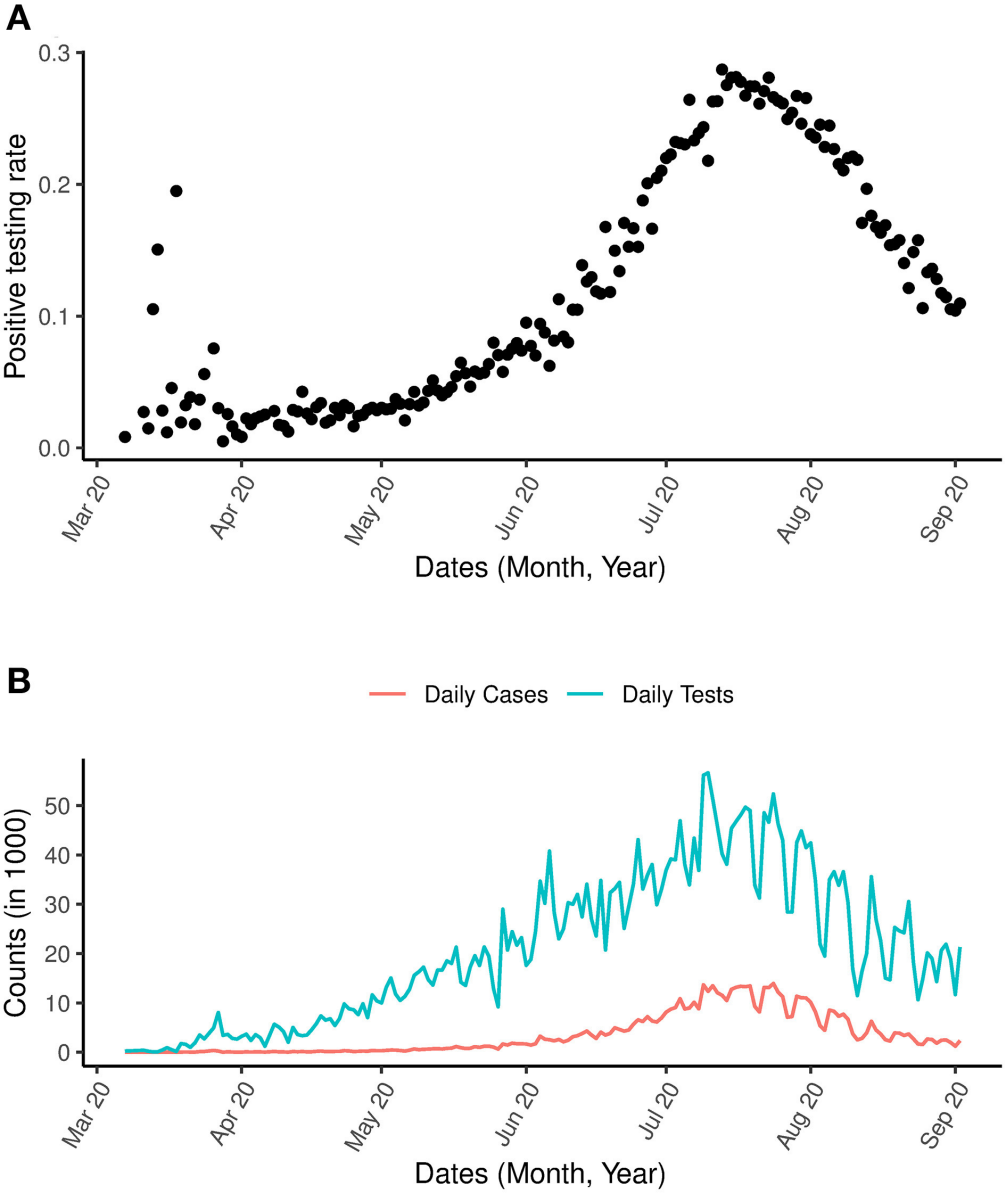
COVID-19 and positive testing rate. **(A)** Is the positive testing rate. **(B)** Is the daily number of cases and daily number of COVID-19 tests between March 7th, 2020 and September 2nd, 2020.

The daily number of reported COVID-19 cases and tests for the period of March 7th, 2020 to September 2nd, 2020 are presented in Figure 2B. The growth of COVID-19 infections in South Africa appears to be tri-phasic especially during the early phase when the cumulative cases were low with rapid growth until March 27th, 2020. A total of 243 new daily cases were observed on March 27, followed by a sharp decline in the rate of new cases. From March 28th, 2020 to April 6th, 2020 the daily increase in cases was consistently below 100. From May 2020 onwards, a consistent increase of more than 1,000 cases per day was observed. The first peak period was between July 9th and 22nd 2020 where more than 10,000 cases were reported on a daily basis. As of July 2020, a total of 3,726,721 tests had been conducted, corresponding to a testing rate of 22.816 per 1,000 population. Throughout this period, the proportion of infections increased until mid July when it started to decrease (Figure 2A).

**Figure 3:**
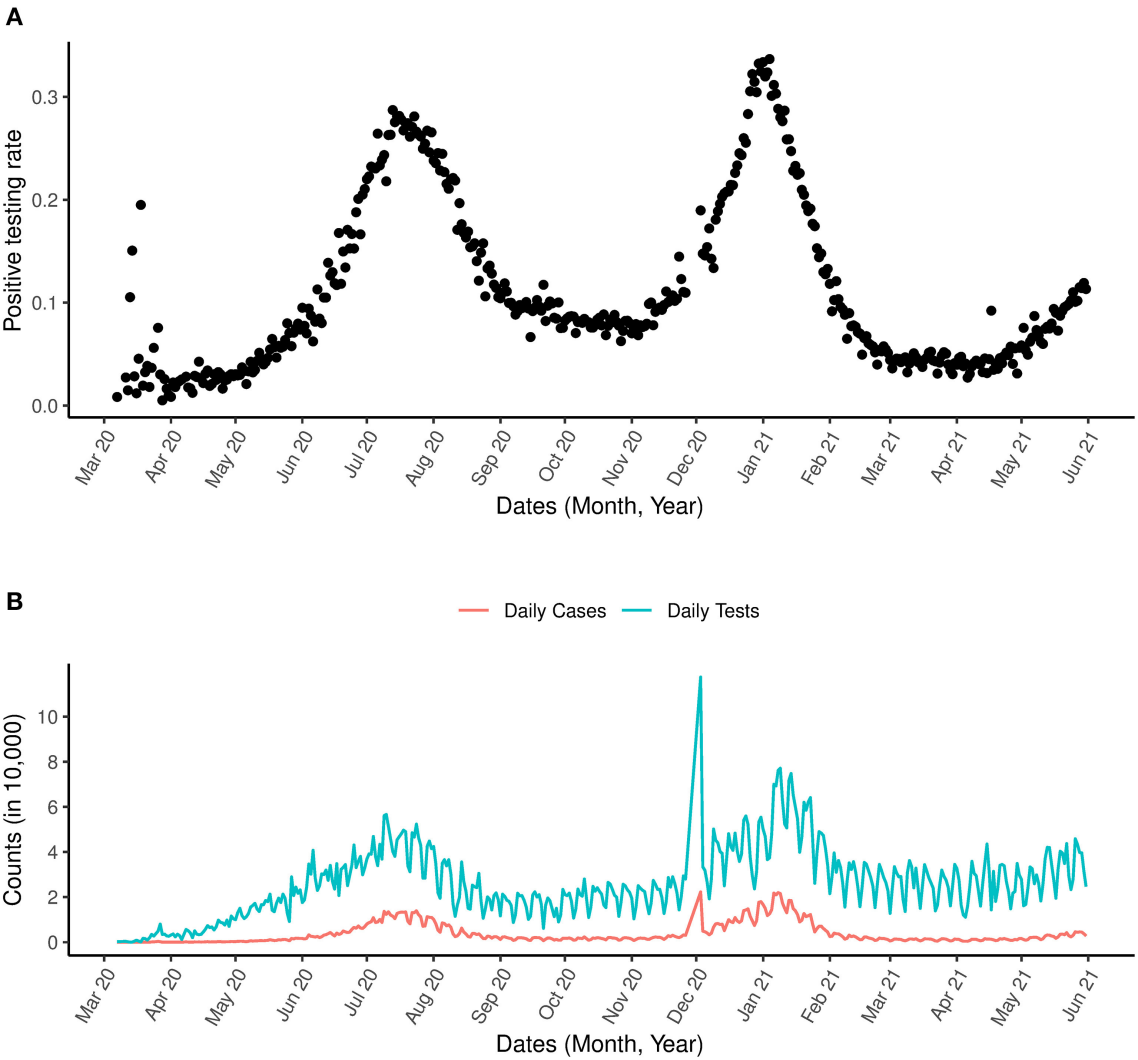
COVID-19 and positive testing rate. **(A)** Is the positive testing rate. **(B)** Is the daily number of cases and daily number of COVID-19 tests between March 1st, 2020 and May 31st, 2021.

In addition to the analysis applied to the data above, which is zoomed in on the first wave of the outbreak, we present also analysis for the most updated data for the period between March 7th, 2020 and May 31st, 2021 incorporating the second wave in Figure 3. An indication for a possible third wave is seen in Figure 3A as an increase in the positive testing rate was observed from May 2021. A sharp spike in the number of COVID-19 tests was observed in 2020 (Figure 3B).

#### 2.1.2. Testing policy in South Africa within the first wave period

A total of 3,245,087 tests for SARS-CoV-2 were conducted between March 1st and August 29th 2020. These tests were performed on individuals who satisfied the case definition for persons under investigation (PUI). The data we used for the analysis presented in this paper were obtained from the COVID19 R package by Guidotti and Ardia (33) which is publicly and continuously updated. For the analysis presented in this paper data until May, 31, 2020 were included. The PUI definition, which was amended consistently included at least one of the following criteria: symptomatic individuals seeking testing, hospitalized individuals for whom testing was done, individuals in high-risk occupations (e.g., health care workers), individuals in outbreak settings, and individuals identified through community screening and testing programmes which were implemented between April 2020 and the middle of May 2020. The number of tests performed on a weekly basis increased from March 2020 until the third week of May 2020, and proceeded by a decrease over the subsequent 2 weeks due to a limited supply of testing kits. The average time elapsed from specimen collection to testing was under 2 days in both the private and public sectors from August 22th to August 29th, 2020.

### 2.2. Methods

#### 2.2.1. Modeling COVID-19 infection rate in South Africa using generalized linear mixed effects model for binary data

##### 2.2.1.1. Model Formulation for the Positive Testing Rate

The number of positive cases among the number of tests is assumed to be binomially distributed. Let π_*t*_ be the daily positive testing rate per test, *Y*_*t*_ be the daily number of COVID-19 cases and *n*_*t*_ be the daily number of COVID-19 tests. Our aim is to model the probability π_*t*_ and to produce a model-based estimate for its first derivative, i.e., the change in the positive testing rate over time. Semi-parametric regression model for binomial data was used to provide an estimate of the positive testing rate as a function of time. The relationship can be expressed as


(1) $$\begin{array}{l} Y_{t}\sim \text{Bin}\left(n_{t},\pi_{t}\right),\;t=1,\ldots ,T,\\ \text{logit}\left(\pi_{t}\right)=f\left(t\right). \end{array}$$

Here, *f*(*t*) is a smooth function of the time *t*. Smoothing splines are commonly used for this purpose (34). A general spline model of degree *d* with *K* knots can be written as follows:


(2) $$\begin{array}{l} \text{logit}\left(\pi_{t}\right)=\beta_{0}+\beta_{1}x_{i}+\cdots +\beta_{d}x_{i}^{d}\\ \;+\overset{K}{\underset{k=1}{\sum }}u_{k}s_{k}\left(x_{i}\right),\;i=1,\ldots ,n\text{ and }k=1,\ldots K, \end{array}$$

Where *s*_*k*_(*x*) is a set of spline basis functions and *u*_*k*_ are a set of random effects that are discussed below.

To avoid overfitting, the spline model is typically estimated by considering penalized maximum likelihood estimation, with a penalty term of the form $\lambda \underset{k}{\sum }u_{k}^{2}$. Ruppert et al. (34) showed that the penalized regression model formulated in Equation (2) can be expressed as a generalized linear mixed effects model (GLMM) given by:


(3) $$\begin{array}{l} \text{logit}\left(\pi \right)=X\beta +\text{Z}\text{u}, \end{array}$$

With $\pi ={\left[\pi_{1},\pi_{2},\ldots ,\pi_{T}\right]}^{T}$, $\beta ={\left[\beta_{0},\beta_{1},\ldots ,\beta_{d}\right]}^{T}$, and $\text{u}={\left[{\text{u}}_{1},{\text{u}}_{2},\ldots ,{\text{u}}_{\text{K}}\right]}^{\text{T}}$. Note that **β** and **u** are vectors of the fixed and random effects, respectively, with $u_{k}\sim N\left(0,\sigma_{u}^{2}\right)$ where $\sigma_{u}^{2}$ acts as the smoothing parameter. This representation has the advantage that the degree of smoothing can be estimated from the data using standard mixed-model software (e.g., Ruppert et al. (34), chapter 4). The design matrices **X** and **Z** are defined as follows:


$$\text{X}=\left[\begin{array}{cccc} 1 & x_{1} & \ldots & x_{1}^{d}\\ 1 & x_{2} & \ldots & x_{2}^{d}\\ \vdots & \vdots & \ddots & \vdots \\ 1 & x_{T} & \ldots & x_{T}^{d} \end{array}\right],$$

and


$$\text{Z}=\left[\begin{array}{cccc} s_{1}\left(x_{1}\right) & s_{2}\left(x_{1}\right) & \ldots & s_{K}\left(x_{1}\right)\\ s_{1}\left(x_{2}\right) & s_{2}\left(x_{2}\right) & \ldots & s_{K}\left(x_{2}\right)\\ \vdots & \vdots & \ddots & \vdots \\ s_{1}\left(x_{T}\right) & s_{2}\left(x_{T}\right) & \ldots & s_{K}\left(x_{T}\right) \end{array}\right].$$

The estimation of the model formulated in Equation (3) is performed by means of penalized quasi-likelihood (PQL). Initial estimates for **β** and **u** are used to calculate the pseudo-data y^*^:


(4) $$\begin{array}{l} {\text{y}}^{*}=X\beta +Zu+W^{-1}\left(\text{y}-\pi \right)\equiv X\beta +Zu+\varepsilon^{*}, \end{array}$$

Where ***W*** is a diagonal matrix with variances of *y*_*t*_ on the diagonal. The pseudo-error ε^*^ has a variance-covariance matrix ***R*** = ***W***^−1^ϕ, where ϕ is the dispersion parameter, equal to one for the standard binomial model family. Equation (4) resembles a LMM formulation for y^*^. Thus, a inear mixed model (LMM) is fitted to the pseudo-data, yielding updated estimates of **β**, ***u***, $\sigma_{u}^{2}$, and ϕ. The procedure of calculating pseudo-data and re-fitting the LMM is repeated until convergence.

#### 2.2.2. Estimating the change in the positive testing rate

To understand the change in the positive testing rate over time, we propose to estimate the rate of change in the positive testing rate over time using the derivative of π_*t*_ given by


(5) $$\begin{array}{l} \pi_{t}^{\prime }=\frac{\pi_{\left(t\right)}-\pi_{\left(t-1\right)}}{\Delta \left(t\right)}. \end{array}$$

Note that is assumed that if the number of tests is constant over time and applied to a random sample of the population, $\pi_{t}^{\prime }$ can give an indication to the change in the virus transmission in the population (since in this case, it is gives the change in transmission probability). However, it is unlikely to assume that the number of tests will be constant nor that the tests will be applied to random sample from the population. However, even in this case, the derivative provides a good indication about the general trend of the virus' transmission for the tested population and can be used as a tool to assess the success of an implemented intervention strategy.

#### 2.2.3. Construction of pointwise confidence band

According to Ruppert et al. (34), an approximate 100(1-α)% pointwise confidence band for an estimated penalized spline in the GLMM framework, $\hat{f}\left(x\right)$, is given by:


(6) $$\begin{array}{l} \hat{f}\left(x\right)\pm z_{1-\alpha /2}\times \hat{\text{st}.\text{dev}}\left\{\hat{f}\left(x\right)-f\left(x\right)\right\}, \end{array}$$

where


(7) $$\begin{array}{l} \hat{\text{st}.\text{dev}}\left\{\hat{f}\left(x\right)-f\left(x\right)\right\}=\sqrt{C_{x}\hat{Q}C_{x}^{T}}, \end{array}$$

with $C_{x}=\left(1\;x\;\ldots \;x^{d}\;s_{1}\left(x\right)\;\ldots \;s_{K}\left(x\right)\right)$ and


(8) $$\begin{array}{l} \hat{Q}=\hat{\text{cov}}\left[\begin{array}{c} \hat{\beta }\\ û-u \end{array}\right]={\left(C^{T}{\hat{R}}^{-1}C+1/{\hat{\sigma }}_{u}^{2}D\right)}^{-1}, \end{array}$$

where,


$$C=\left[\begin{array}{cccc} 1 & x_{1} & \ldots & x_{1}^{d}\\ 1 & x_{2} & \ldots & x_{2}^{d}\\ \vdots & \vdots & \ddots & \vdots \\ 1 & x_{T} & \ldots & x_{T}^{d} \end{array}\right]\;[\begin{array}{cccc} s_{1}\left(x_{1}\right) & s_{2}\left(x_{1}\right) & \ldots & s_{K}\left(x_{1}\right)\\ s_{1}\left(x_{2}\right) & s_{2}\left(x_{2}\right) & \ldots & s_{K}\left(x_{2}\right)\\ \vdots & \vdots & \ddots & \vdots \\ s_{1}\left(x_{T}\right) & s_{2}\left(x_{T}\right) & \ldots & s_{K}\left(x_{T}\right) \end{array}]$$

and $D\equiv \text{diag}\left(\left[0_{d+1}^{T},1_{K}^{T}\right]\right)$

Pointwise confidence bands, however, need to be corrected for multiplicity. In addition, they ignore serial correlation. Therefore, we make use of simultaneous confidence bands implemented in Claesen et al. (35), which allow to make joint statements on multiple locations of the fitted curve. A 100(1-α)% simultaneous confidence band for ${\hat{f}}_{x}$ is defined as:


(9) $$\begin{array}{l} {\hat{f}}_{x}\pm c_{1-\alpha }\times \hat{\text{st}.\text{dev}}\left\{\hat{f}\left(x\right)-f\left(x\right)\right\} \end{array}$$

Where the critical value, *c*_1−α_, is the (1- α) quantile of the random variable


$$\text{su}{\text{p}}_{\text{x}\in \text{χ}}|\frac{\hat{f}\left(x\right)-f\left(x\right)\}}{\hat{\text{st}.\text{dev}}\left\{\hat{f}\left(x\right)-f\left(x\right)\right\}}|\approx \underset{1\leq l\leq M}{\max}|\frac{{\left(C_{x}\left[\begin{array}{c} \hat{\beta }-\beta \\ \hat{\text{u}}-\text{u} \end{array}\right]\right)}_{l}}{\hat{\text{st}.\text{dev}}\left\{\hat{f}\left(x_{l}\right)-f\left(x_{l}\right)\right\}}|,$$

Which can be found by simulating from an approximate multivariate normal distribution (34):


$$\left[\begin{array}{c} \hat{\beta }-\beta \\ \hat{u}-u \end{array}\right]\sim N\left\{\begin{array}{c} 0,\hat{\text{Q}} \end{array}\right\}.$$

Note that the GLMM formulated in Equation (3) is used to explore the trend in the positive rate evolution over time but not for prediction of the future positive rate outside the range the observed data.

## 3. Results

**Figure 4:**
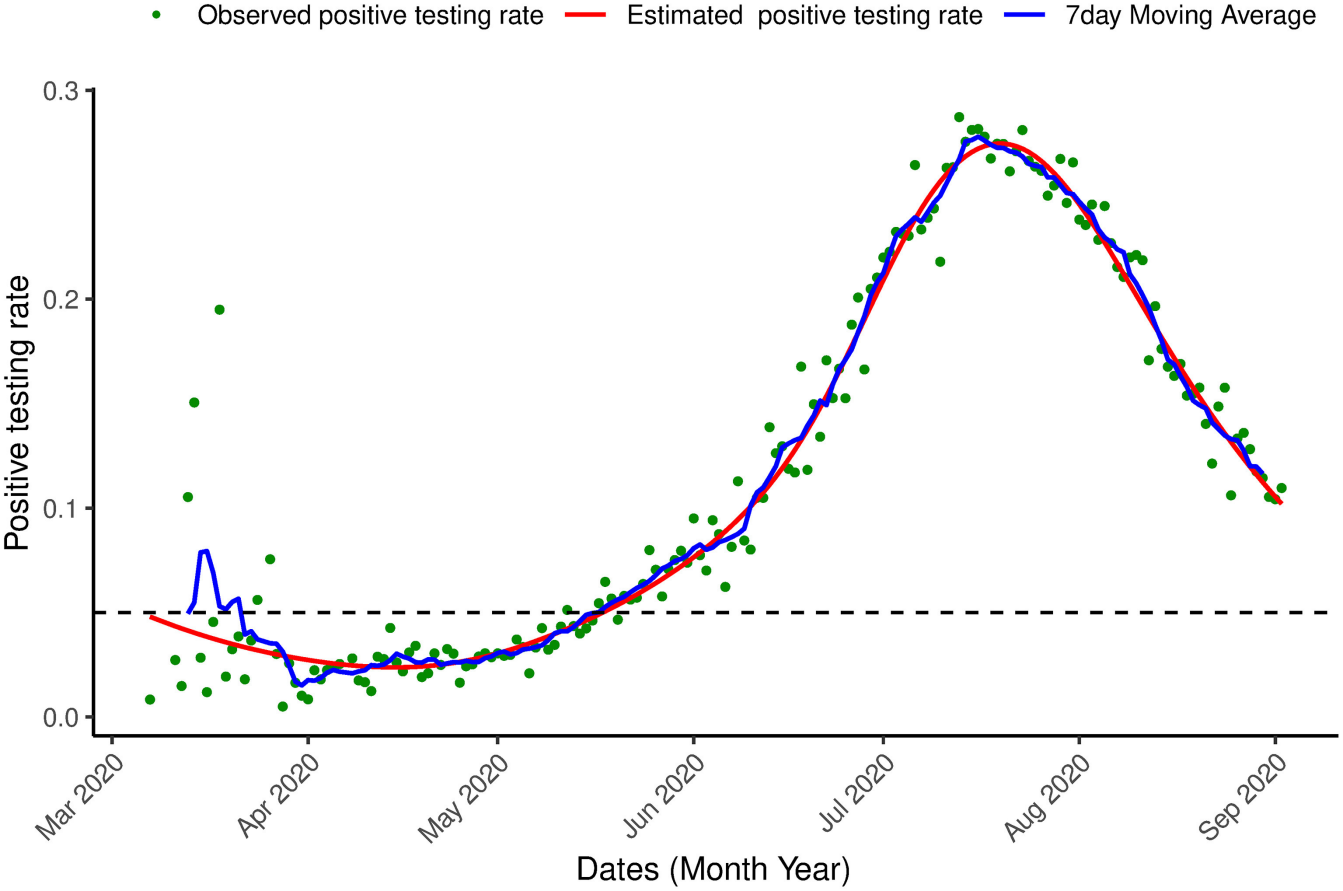
Proportion of infection. Positive testing in South Africa. Observed positive testing rate over time, estimated positive testing rate (red line) and a 7 day moving average (blue line) between March 7th, 2020 and September 2nd, 2020.

A generalized additive model was fitted to the data with the time component as the smooth term using the gam() function of the mgcv library (36) in R (37). The model was first applied to the data of the first wave of the outbreak (March, 7th 2020-September, 2nd 2020). Figure 4, shows that the estimated positive testing rate reached its peak on July, 21st 2020, at the same time that the number of tests was at its highest level. From that time onward, both number of tests and the positive testing rate declined. This could be a result of a reduction of the virus' transmission in the population or a result of a change in the population to which the tests were applied.

**Figure 5:**
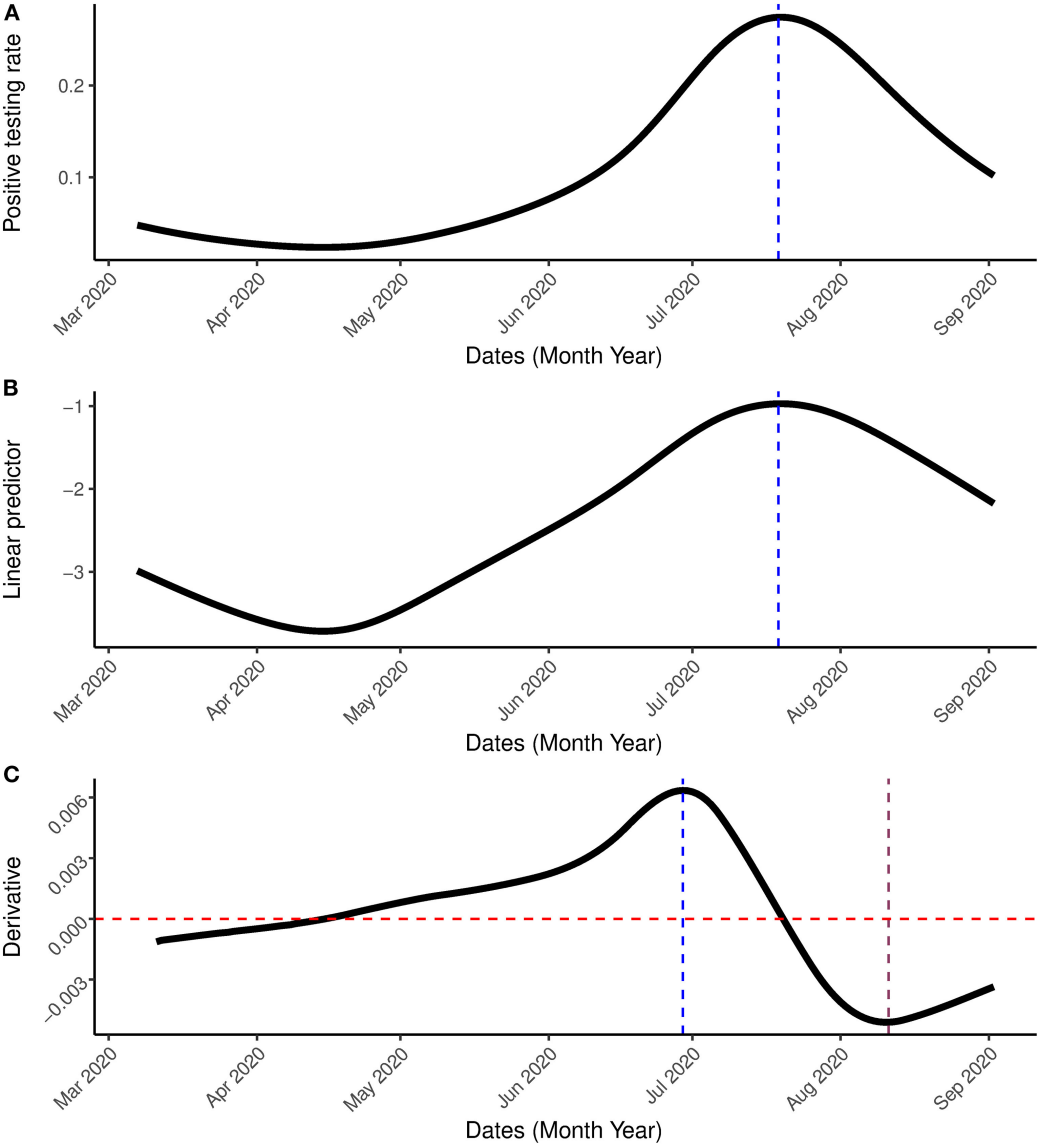
Result incorporating the first wave. **(A)** Is the estimated positive testing rate with 95% simultaneous confidence band. **(B)** Is the linear predictor of the smoother with 95% simultaneous confidence band. **(C)** Is the first order derivative of the estimated probability with 95% simultaneous confidence band between March 7th, 2020 and September 2nd, 2020. The blue vertical line represents the peak, the pink vertical line represents the date in which the rate of change started to increase while the red horizontal line represents the rate of change at 0.

From July 19th 2020 onward, the change in positive testing rate (the derivative plot presented in lower panel of Figure 5) is negative (indicating a decline in the positive testing rate) but from August, 21st, 2020, the derivative begins to increase (although it is still negative). This suggests a change in the transmission trend and gives an indication for a possible increase in the number of positive cases in the near future. Indeed, such a increase was observed on October 18th, 2020 (see the analysis below).

**Figure 6:**
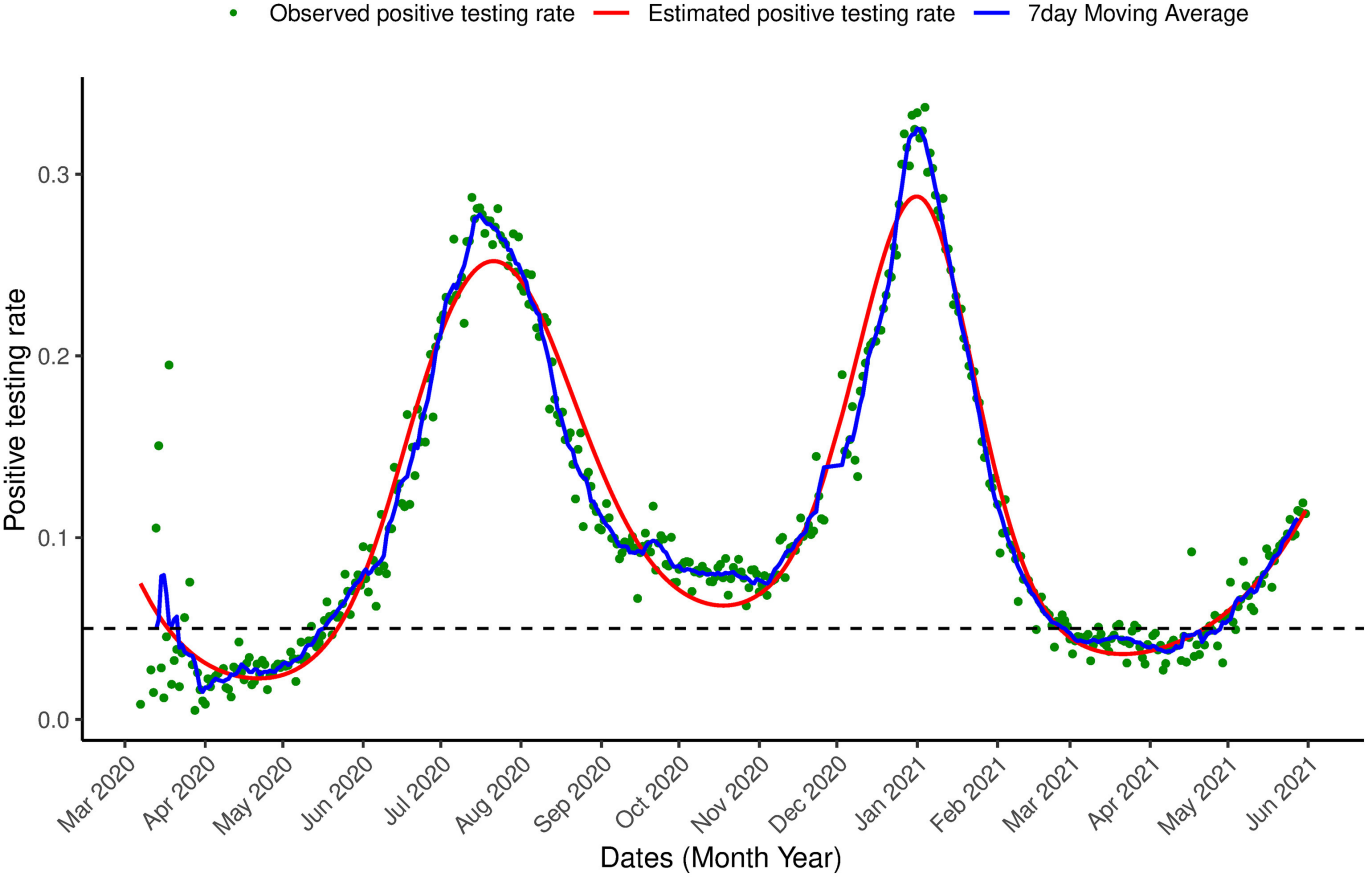
Proportion of infection Positive testing in South Africa. Observed positive testing rate over time, estimated positive testing rate (red line) and a 7 day moving average (blue line) between March 7th, 2020 and May 31st, 2021.

**Figure 7:**
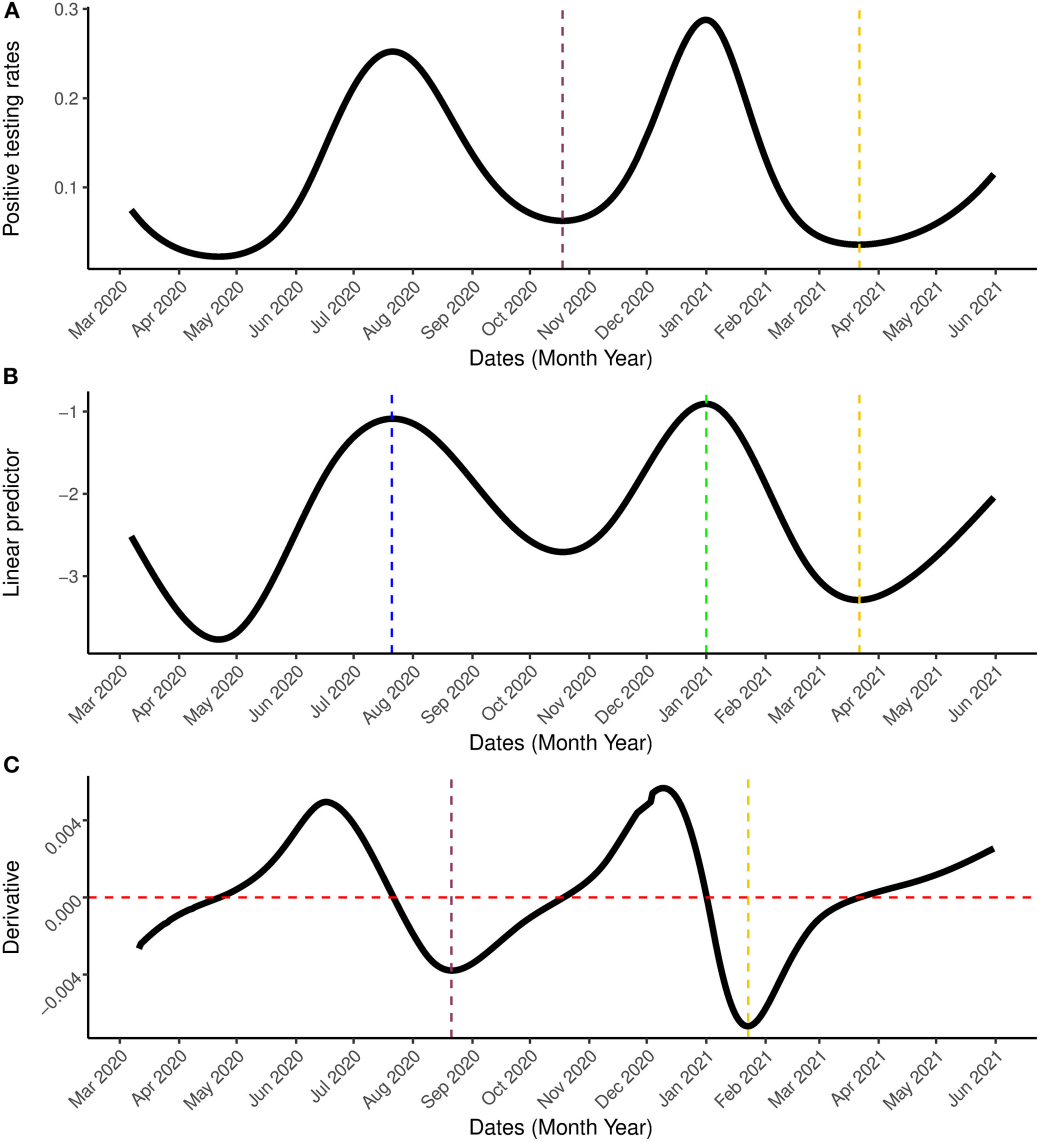
Result incorporating the second wave. **(A)** Is the estimated positive testing rate with 95% simultaneous confidence band. **(B)** Is the linear predictor of the smoother with 95% simultaneous confidence band. **(C)** Is the first order derivative of the estimated probability with 95% simultaneous confidence band between March 7th, 2020 and May 31st, 2021. The blue and green vertical lines represents the peak periods while the pink and gold vertical lines are the turning points. In **(C)**, the gold line represents the date on which the rate of change started to increase (January 23rd, 2021) while the red horizontal line represent the rate of change at 0.

Next, the model was applied to the most updated dataset that was available when the paper was written (March 7th, 2020–May 31st, 2021) which contains information on the first two waves of the outbreak observed in South Africa and the beginning of a (possible) third wave. Figures 6, 7 show the estimation for the positive testing for the updated data. As shown above, the positive testing was first peaked on July, 21st 2020 [$\hat{\pi }$ = 0.252, C.I. = (0.251,0.263)], later decreased, and a second peak was observed on January 1st, 2021 [$\hat{\pi }$ = 0.288, C.I. = (0.287, 0.288)]. Figure 7 shows the estimated positive rate (Figure 7A) and its corresponding first derivative (Figure 7C). We notice that the first turning point in the first derivative curve was observed on August 21st, 2020. On this day, the derivative began to increase (although still negative) while the positive testing rate continued to decrease illustrating that the model based first derivative was able to give a clear indication 2 months prior of the increase of the positive rate (that was observed on October 18th, 2020). Similar pattern was observed in the third wave. The second turning point of the first derivative was observed on January 23rd, 2021, which gives an indication that South Africa might face a third outbreak which indeed was observed from March 23th, 2021 onward.

## 4. Discussion

In view of the existing healthcare challenges in South Africa and other parts of the world, reliable and accurate knowledge about the positive testing rate of COVID-19 is important to ensure optimal resource allocation and better understanding of the transmission process. It is important to note that the method we propose in this paper was developed as an exploratory tool that different users can use to produce similar output for their countries using publicly available datatset with limited information. This ensures that the current situation of the COVID-19 outbreak in a country/province can be reported almost in real-time without the need to wait for official permission from the government. This is useful in countries for which the government is reluctant to release the current information about the COVID-19 outbreak. As an exploratory tool, the model is estimated within the range of the data and we do not aim to predict the positive rate outside the range of the available data. Therefore, we do not split the data into training and testing datasets as done when prediction is of primary interest. When we developed our modeling approach, we focused on three main concepts: (1) using the positive testing to get an insight into the epidemic evolution (2) usage of publicly available data with limited information, and (3) implementation of the proposed model as a dashboard for which the code (in R) is publicly available. These three concepts ensure that different readers/users will be able to produce a similar output for their countries.

In the current study we modeled the COVID-19 cases out of the number of tests as a function of time using a semi-parametric approach. This approach allows us to take into account the number of tests performed, which when ignored, might lead to erroneous conclusions. The model allows us to overcome the problem of modeling the number of cases alone and to take into account the strong positive relationship between the number of cases and the number of tests. As this can lead to misleading results and therefore affect government policy regarding measures and precautions needed.

The positive testing rate decreased from early March 2020 when the disease was first observed until early May 2020 after which it kept on increasing. In July 2020, the infection reached its peak and then consistently decreased, indicating that restrictions and lockdowns meant to slow down. From mid August, 2020, the rate of change of the positive testing rate indicates that the decline in the positive testing rate is slowing down suggesting that a less effective intervention is currently implemented and a possible second wave.

Though, in this paper, we focus on South Africa, the method can be used for other countries with any vaccination and testing programme. Examples for four different countries, Ethiopia and India with a low rate of vaccination coverage (1.58 and 12.12% respectively, May 31st, 2021), Poland with a moderate vaccination coverage (36.22%, May 31st, 2021) and United Kingdom with high rate of vaccination coverage [58.15%, May 31st, 2021 (38)] are presented in Supplementary material. The method proposed in this paper was implemented in an R dashboard for all the countries with publicly available data in the COVID-19 R package data. The dashboard presents the outbreak data and uses the proposed method to estimate and visualize the positive testing probability for any selected country. The R code for the method and data analysis can be accessed in the code section of the dashboard which can be downloaded for free using the following link: COVID19dashboardMC.

Our analysis of the updated dataset has revealed the possibility of a third wave in South Africa. An indication of the possibility for the third wave was already detected by the proposed model on January 23rd, 2021, 60 days before the positive testing rate started to increases on March 22nd, 2021 (see Figure 7). This could help the government in the preparation and implementation of interventions for COVID-19.

The model applied is based entirely on the observed data at hand. The ability to use the model based derivative to predict an outbreak few weeks before it occurs is a powerful approach for understanding and learning the outbreak of various countries using only publicly available datasets. The main advantage of this approach is that it allows to model COVID-19 outbreak without the need of getting consent from the government to use official information such as the disease spread, sampling and tracing information. In many low and income countries, this type of information is typically release in a long time delay and introduced bias in modeling. While other models, such as the compartmental models, may be appealing in their ability to examine various scenarios and estimate impact of possible interventions, these models are heavily dependent on assumptions. The compartmental modeling approach was applied to forecast COVID-19 cases and deaths in South Africa and was used to guide the government on public health interventions. There was however, substantial uncertainty in these predictions, and the assumptions governing these models were criticised by Muller (39) and Consortium (40). In conclusion, the results obtained from our model need to be interpreted under the background information (if available) of changing COVID-19 testing strategies in the country. When the positive testing rate is tracked in real time, it can provide useful guidance to policy makers as it can provide a useful insight on the current and future trend of the COVID-19 epidemic.

The method we propose was developed as an exploratory tool that different users can use to produce similar output for their countries using publicly available datatset with limited information. This ensures that the current situation of the COVID-19 outbreak in a country/province can be reported almost in real-time without the need the wait for official permission from the government. This is useful in countries for which the government is reluctant to release the current information about the COVID-19 outbreak. As an exploratory tool, the model is estimated within the range of the data and we do not aim to predict the positive rate outside the range of the available data. Therefore, we do not split the data into training and testing datasets as done when prediction is of primary interest. When we developed our modeling approach, we focused on three main concepts: (1) using the positive testing to get an insight into the epidemic evolution (2) usage of publicly available data with limited information, and (3) implementation of the proposed model as a dashboard for which the code (in R) is publicly available. These three concepts ensure that different readers/users will be able to produce a similar output for their countries. The proposed model was developed as an exploratory tool that allows the users to get an insight into the disease evolution in their countries. Specifically, one strength of this modeling approach is the lack of dependence on assumptions regarding the transmission process.

## Data availability statement

Publicly available datasets were analyzed in this study. This data can be found at: https://github.com/owid/covid-19-data/tree/master/public/data.

## Author contributions

OO wrote the initial draft, took the lead in writing the manuscript, and performed the analysis. SM, TR, and ZS contributed to the design and implementation of the research and the writing of the manuscript. JC provided materials on methodology. JC and AK contributed to various revisions of the manuscript. RSe, RSh, and SS created the R online dashboard. All authors contributed to the final version of the manuscript and approved the final submission of the manuscript.
