# Supplementary material for: Modeling the positive testing rate of COVID-19 in South Africa using a semi-parametric smoother for binomial data
Source: Front Public Health. 2023 Feb 22;11:979230. doi: 10.3389/fpubh.2023.979230 (PMC9992730; doi:10.3389/fpubh.2023.979230)
Supplement: Supplementary file 1 [file Data_Sheet_1.pdf]

Supplementary Appendix: Modelling the Positive Testing Rate of COVID-19 in South Africa Using A Semi-Parametric Smoother for Binomial Data

Olajumoke Evangelina Owokotomo[1*], Samuel Manda[2,3*], Jürgen Claesen[1], Adetayo Kasim[5], Rudradev Sengupta[1,6], Rahul Shome[7], Soumya Subhra Paria[8], Ziv Shkedy[1+] and Tarylee Reddy[2,4+].

[1]Data Science Institute, Center for Statistics, I-BioStat, Hasselt University,
Gebouw B, 3590 Diepenbeek, Belgium.

[2]Biostatistics Research Unit at South African Medical Research Council, South Africa.

[3]Department of Statistics, University of Pretoria, South Africa.

[4]University of KwaZulu-Natal, School of Mathematics, Statistics and Computer Science, South Africa

[5]Department of Anthropology & Durham Research Methods Centre, Durham University, UK.

[6]The Janssen Pharmaceutical Companies of Johnson Johnson, Beerse, Belgium.

[7]Department of Computer Science, Rice University, USA.

[8]School of Mathematics and Statistics, The Open University, UK.

[*]Joint first author,[+]Joint last author

1 Introduction

The supplementary appendix contains additional analyses for other countries that were not included in the main manuscript. Four different countries were considered, 2 countries with low rate of vaccination administration (Ethiopia, India), a country with a mild rate of vaccine administration (Poland), a country with high rate of vaccine administration (United Kingdom). Also, we present the results of an extended analysis for the South African data for the period between 01/04/2020 to 31/05/2021.

In addition, an online R dashboard (Sengupta et al., 2021) was developed to visualize the COVID-19 outbreak and implement the methodology discussed. This allows to estimate and visualize the positive testing rate and the rate of change automatically when the dashboard is activated. This dashboard can easily be used by any country without the rigorous stress of coding. The codes for the method can be seen in the code section of the dashboard.

Datasets that are presented in this appendix are publicly available and were obtained using `COVID19` R package (Guidotti and Ardia, 2020).

2 Exploratory Data Analysis Per Country

2.1 Ethiopia

The number of confirmed cases over time in Ethiopia was relatively low, even when the number of test was increased. Over time, the proportion of positive cases increases to a peak at 05/04/2021 (with 28.6% of positive tests and decreased thereafter (see Figure S1)

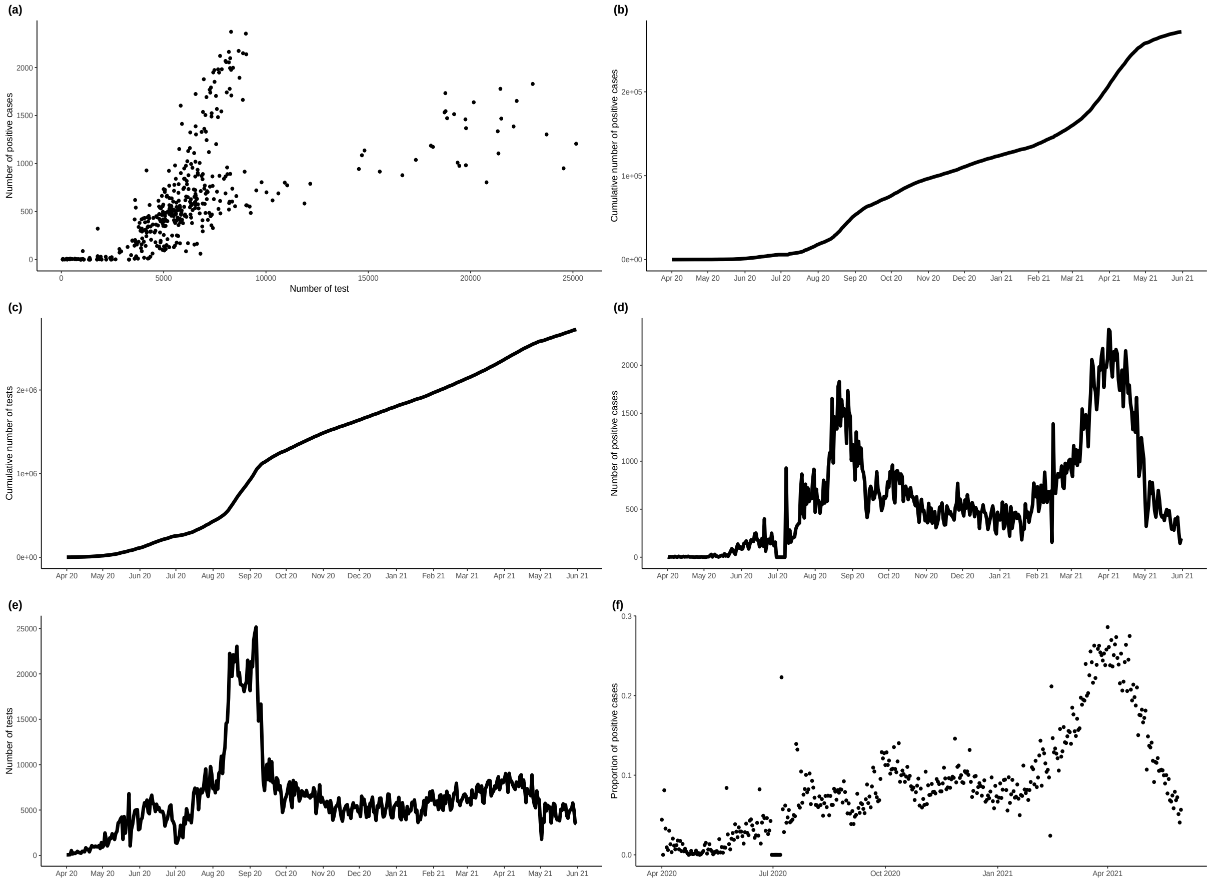

S1. Ethiopia's COVID-19 graphical summary. Panel a: Relationship between the daily number of COVID-19 tests and the daily number of COVID-19 positive cases. Panel b: The cumulative number of COVID-19 cases confirmed over time. Panel c: The cumulative number of COVID-19 tests conducted over time. Panel d: Daily number of COVID-19 cases. Panel e: Daily number of COVID-19 tests. Panel f: The positive testing rate over time.

2.2 Poland

Figure S2 shows that a positive relationship was observed between the number of cases and the number of test (panel a) with, more or less, a fixed number of tests over time (panel e). The proportion of positive tests has the first peak on 25/11/2020 (with 99.7% positive tests) and the

second peak on 23/03/2021 (35% positive tests).

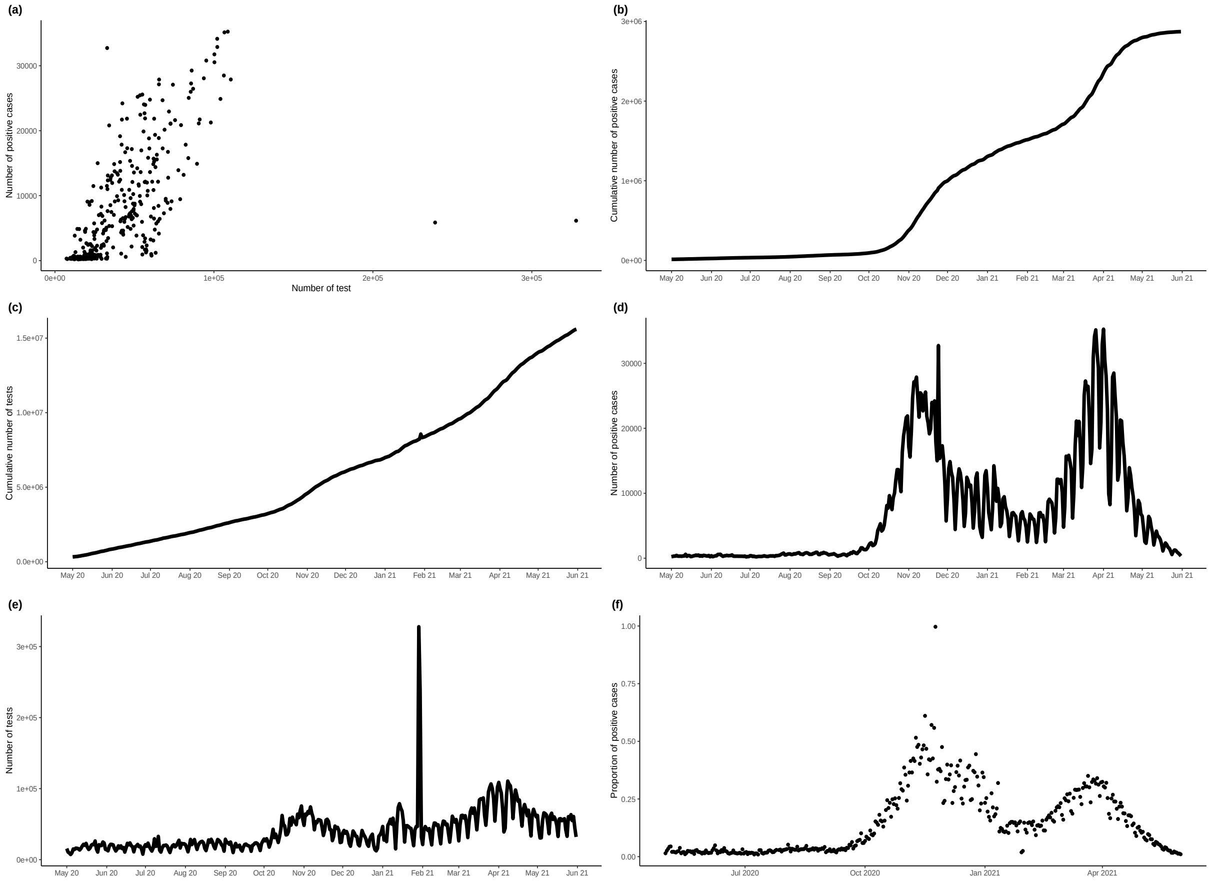

S2. Poland's COVID-19 graphical summary. Panel a: Relationship between the daily number of COVID-19 tests and the daily number of COVID-19 positive cases. Panel b: The cumulative number of COVID-19 cases confirmed over time. Panel c: The cumulative number of COVID-19 tests conducted over time. Panel d: Daily number of COVID-19 cases. Panel e: Daily number of COVID-19 tests. Panel f: The positive testing rate over time.

2.3 United kingdom

In the United Kingdom, the proportion of positive cases was high at the beginning of the outbreak on 05/04/2020 with 46.5% of positive tests, and it reduced over time. A secondary peak is observed at 29/12/2020 with 15.2% of positive tests (see Figure S3) and then reduces over time. This implies a proper management of the COVID-19 pandemic, and currently it is at an all-time low.

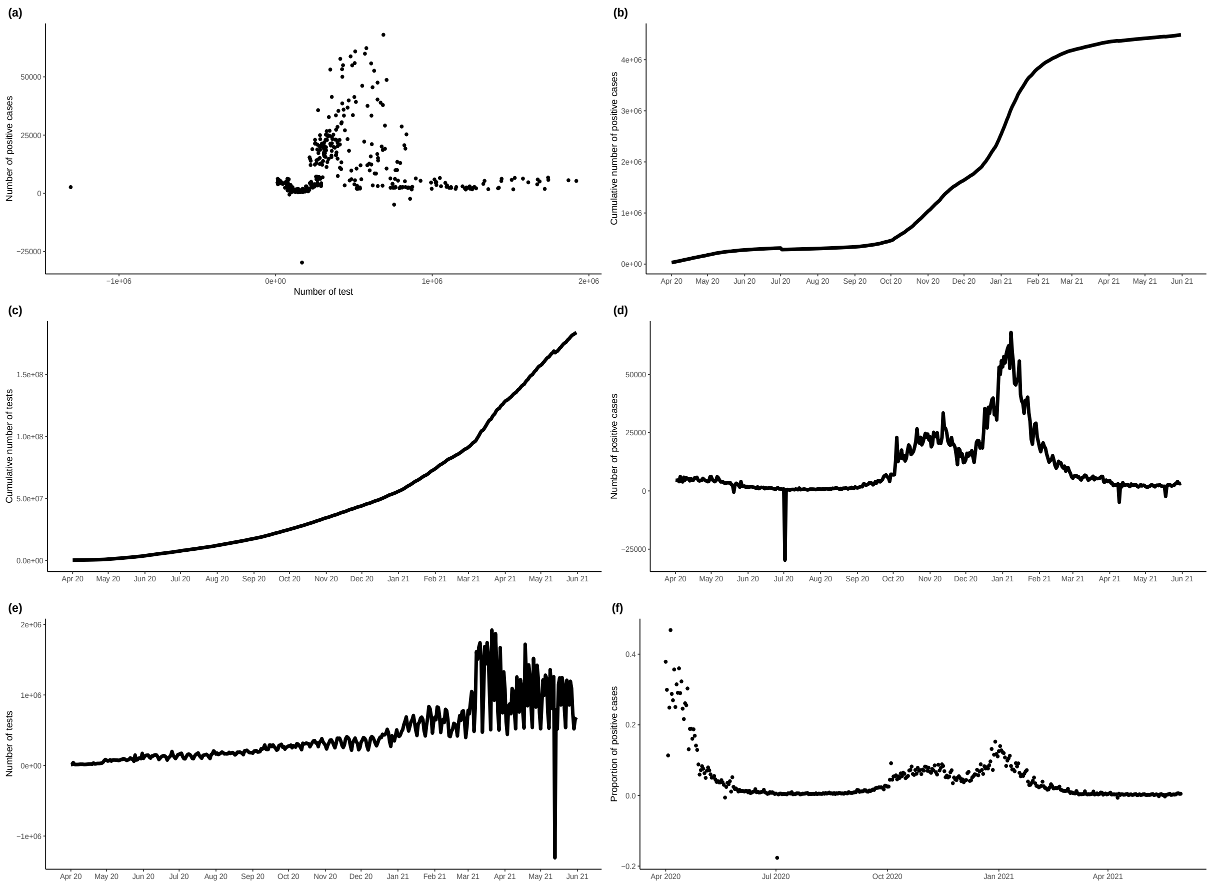

S3. United Kingdom's COVID-19 graphical summary. Panel a: Relationship between the daily number of COVID-19 tests and the daily number of COVID-19 positive cases. Panel b: The cumulative number of COVID-19 cases confirmed over time. Panel c: The cumulative number of COVID-19 tests conducted over time. Panel d: Daily number of COVID-19 cases. Panel e: Daily number of COVID-19 tests. Panel f: The positive testing rate over time.

2.4 India

We consider the COVID-19 outbreak data from India between March 1st, 2020 to July 8th, 2021 (data were downloaded when the dashboard we accessed on July, 8th, 2021). Figure S4 reveals two evident peaks in September 2020 and May 2021 with much more cases in the second peak. During March to May 2020 a nationwide lockdown was imposed. The lockdown was then relaxed in a phased manner, starting from June 2020, when interstate and inter-district movement of people was allowed. This result in an increase in infection rate among adults during the months of August and September 2020. The second wave started in March 2021 followed by a rapid increase in the number of cases in April 2021 onward.

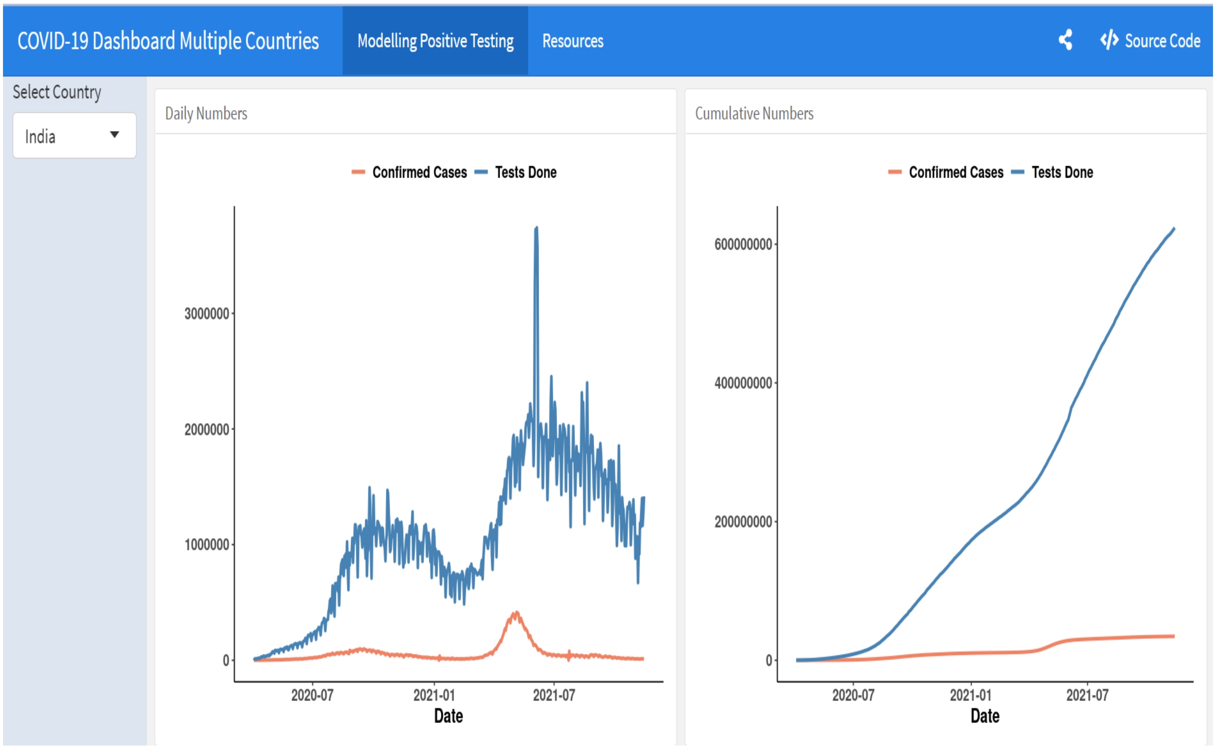

S4. India's COVID-19 data as presented in the dashboard. (https://rdsg.shinyapps.io/COVID19dashboardMC/).

2.5 South- Africa Updated Data

The correlation between the number of cases and the number of tests is positive (see Figure S5) and we can see (panel d and e) that the higher the number of test performed, the higher the number of confirmed cases. The proportion of positive tests has two peaks: the first is observed on 13/07/2020 with 28.7% positive tests and the second on 04/01/2021 with 33.7% of positive tests. The analysis related to this data is presented as the second analysis in the paper.

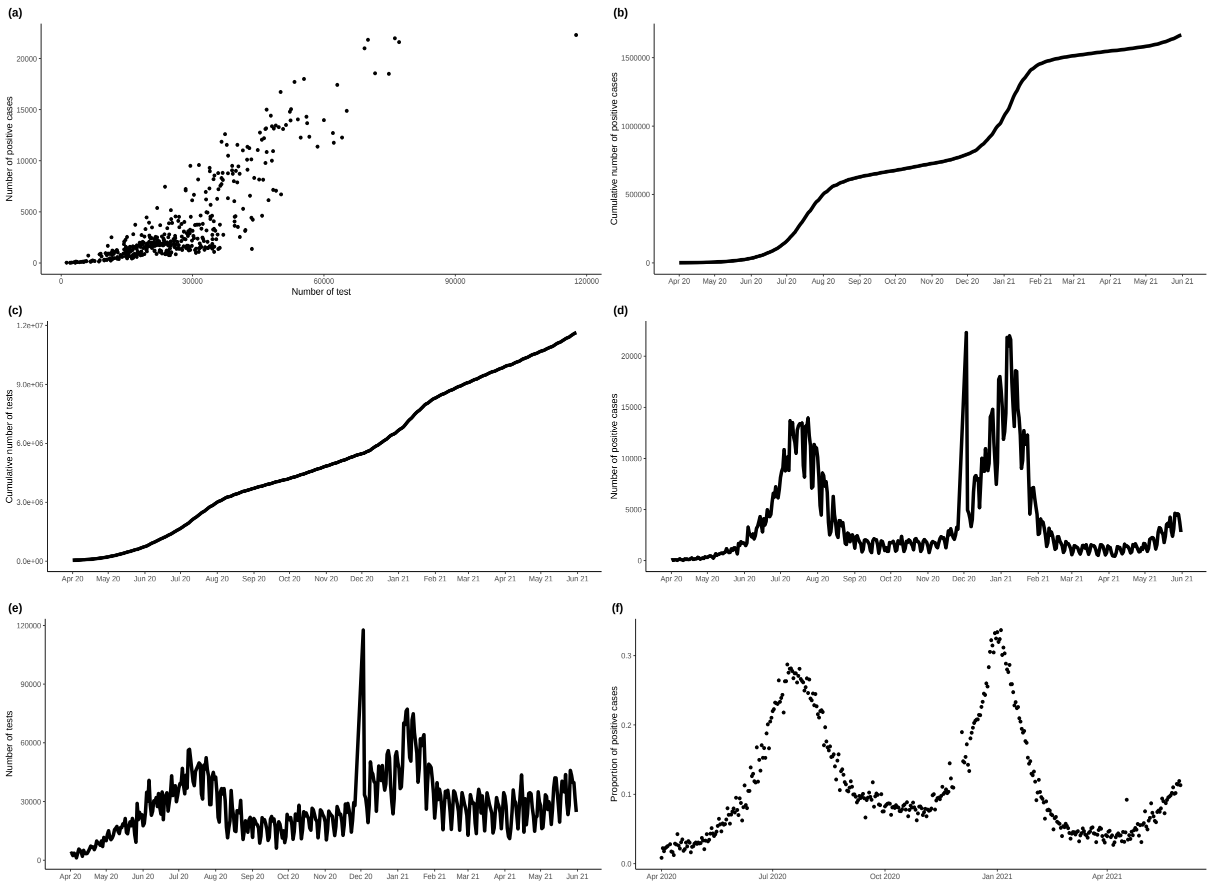

S5. South Africa COVID-19 graphical summary. Panel a: Relationship between the daily number of COVID-19 tests and the daily number of COVID-19 positive cases. Panel b: The cumulative number of COVID-19 cases confirmed over time. Panel c: The cumulative number of COVID-19 tests conducted over time. Panel d: Daily number of COVID-19 cases. Panel e: Daily number of COVID-19 tests. Panel f: The positive testing rate over time.

3 Application to the Outbreak Data

3.1 Ethiopia

The semi-parametric model, formulated in Section 3 (Equation 2) of the manuscript was fitted to the COVID-19 outbreak data in Ethiopia (see Figures S6 and S7). The upper panel in Figure S7 reveal that the probability of positive testing is peaked on 05/04/2021 (=0.251, C.I.=(0.248,0.255)) and decreased thereafter. Note however, that the lower panel in Figure S7 indicates that from 03/05/2021 the rate of change, although negative, start to increase. This implies that the probability of positive testing is either reaches a plateau and stabilized or on the way to a new peak.

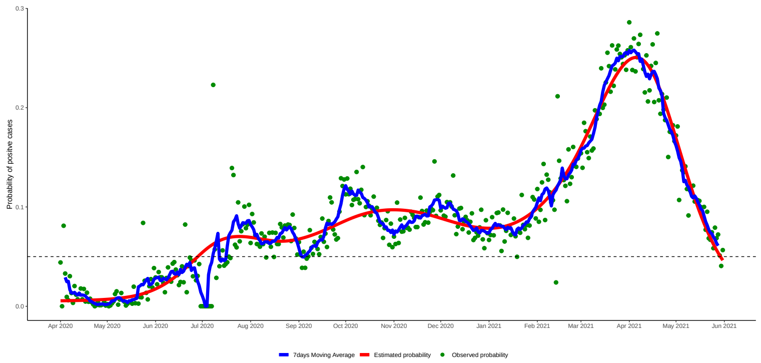

S6. Positive testing rate in Ethiopia. Observed proportion of infection over time, estimated probability (red line) and a 7 days moving average (blue line).

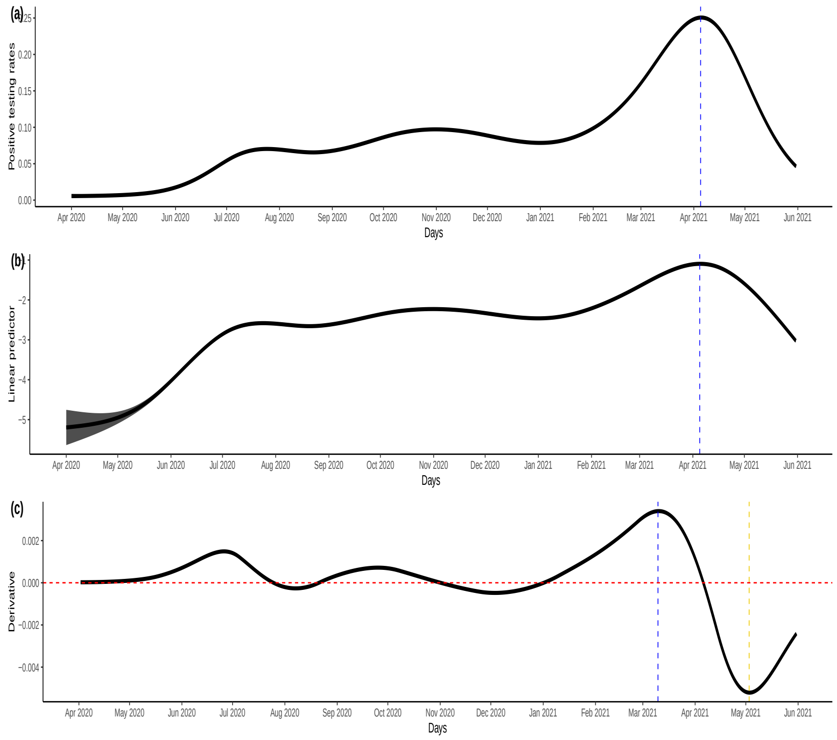

S7. Ethiopia result. Panel a: Estimated positive testing rate with 95% simultaneous confidence band. The blue vertical line represents the peak (April 5th, 2021). Panel b: The linear predictor of the smoother with 95% simultaneous confidence band.The blue vertical line represents the peak (April 5th, 2021). Panel c: The first order derivative of the estimated probability with 95% simultaneous confidence band between March 7th, 2020 and September 2nd, 2020. The blue vertical line represents the peak (March 10th, 2021), the yellow vertical line represent the date in which the rate of changes started to increase (May 3rd, 2021) while the red horizontal line represent the rate of change at 0.

3.2 Poland

Figures S8 and S9 show the results for the positive testing in Poland. Note that after the first peak on 25/11/2020 ($\hat{\pi}$=0.4583, C.I.=(0.457,0.4595)), the probability decreases. The rate of change, presented in the lower panel of Figure S9, indicates that, from 20/12/2020 onward, the probability of positive testing is in the way to a second peak that was indeed observed on 24/03/2021 ($\hat{\pi}$=0.293, C.I.=(0.2937 0.292)).

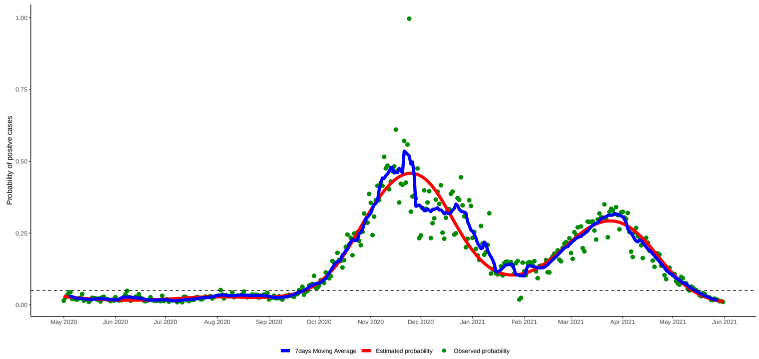

S8. Positive testing rate in Poland. Observed proportion of infection over time, estimated probability (red line) and a 7 days moving average (blue line).

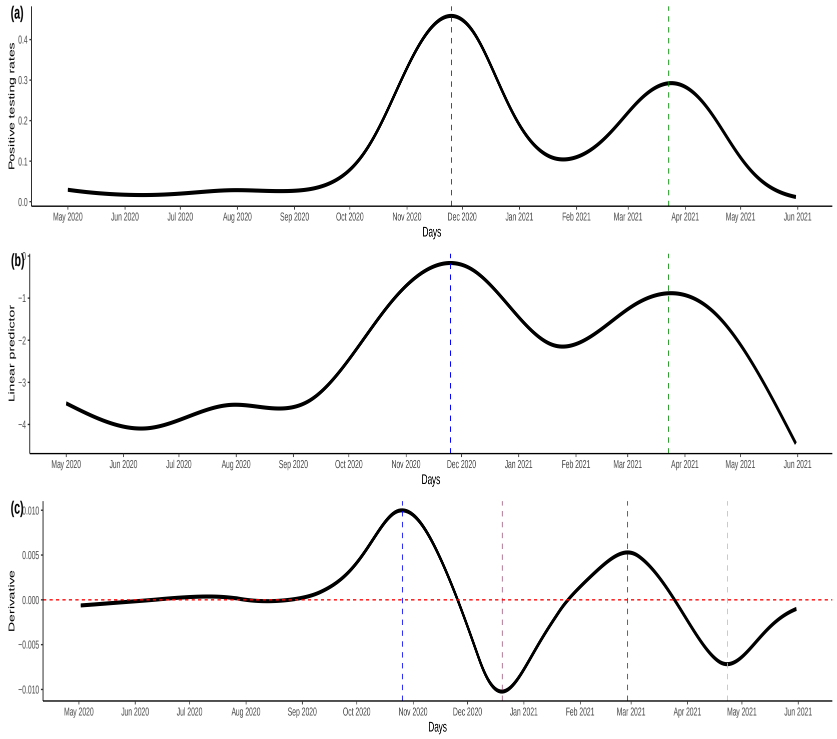

S9. Poland result. Panel a: Estimated positive testing rate with 95% simultaneous confidence band. The blue and green vertical line represents the peaks (November 25th, 2020 and March 23rd, 2021). Panel b: The linear predictor of the smoother with 95% simultaneous confidence band The blue and green vertical line represents the peaks (November 25th, 2020 and March 23rd, 2021). Panel c: The first order derivative of the estimated probability with 95% simultaneous confidence band between March 7th, 2020 and September 2nd, 2020. The blue and green vertical line represents the peaks (October 26th, 2020 and Febuary 27th 2021), the pink and yellow vertical line represent the date in which the rate of changes started to increase (December 20th, 2020 and April 23th, 2021) while the red horizontal line represent the rate of change at 0.

3.3 India

The models presented in the method section of the paper was implemented as a part of the R Shiny online App tool, (Sengupta et al., 2021) which is available online at

`https://rdsg.shinyapps.io/COVID19dashboardMC/`

The `COVID19dashboardMC` dashboard is developed to visualize the trend over time of an outbreak in a country. Selecting India in the country panel of the dashboard will produce Figure S10. The probability of positive testing and its first derivative are implemented as a part of the tool. The dashboard was developed using the publicly available R software, and the code for the methodology is available in the code section of the dashboard.

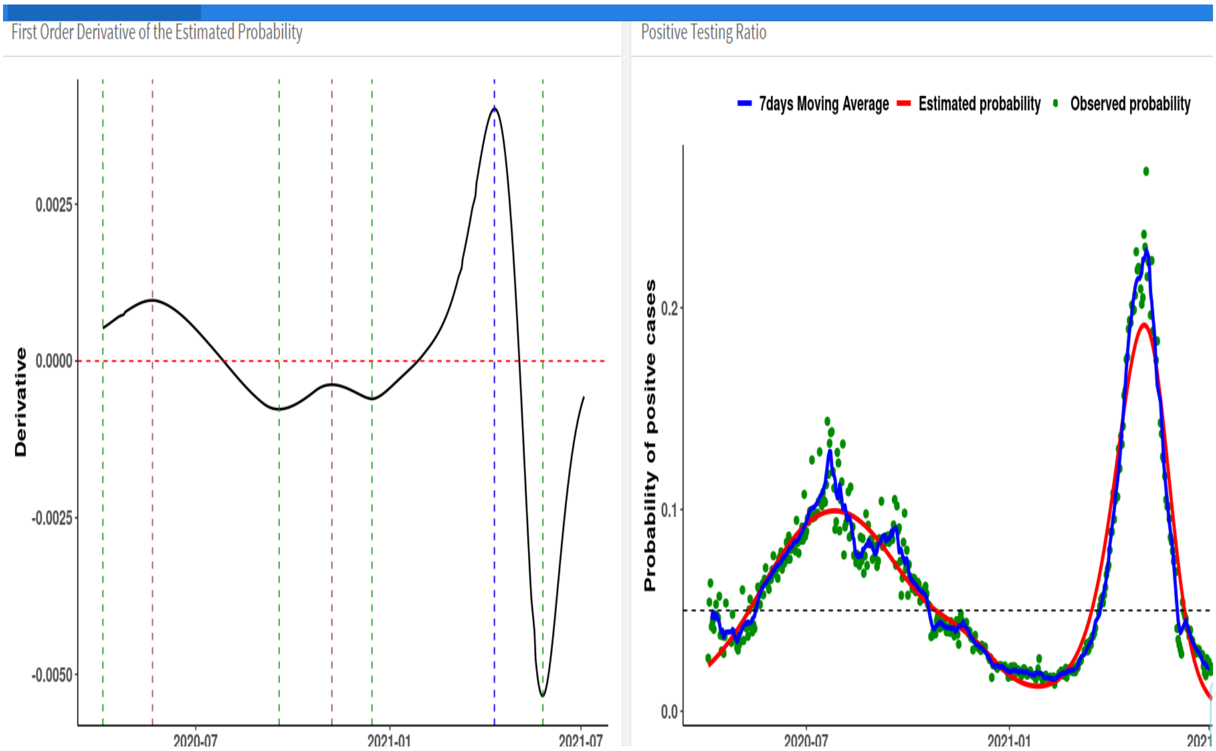

S10. COVID-19 online dashboard. The probability of positive testing (right panel) and its first derivative (left panel) as presented in the online dashboard for the COVID-19 outbreak in India.

3.4 United Kingdom

Figures S11 and S12 show the results for the positive testing in the United Kingdom. The positive testing was peaked at the beginning on 01/04/20 ($\hat{\pi}$=0.423, C.I.=(0.419,0.427)), then the probability decreases. The rate of change, presented in the lower panel of Figure S12, indicates that, from 22/01/2021 onward, the probability of positive testing stabilized and become constant over time as the derivative in the lower panel of Figure S12 converges to zero.

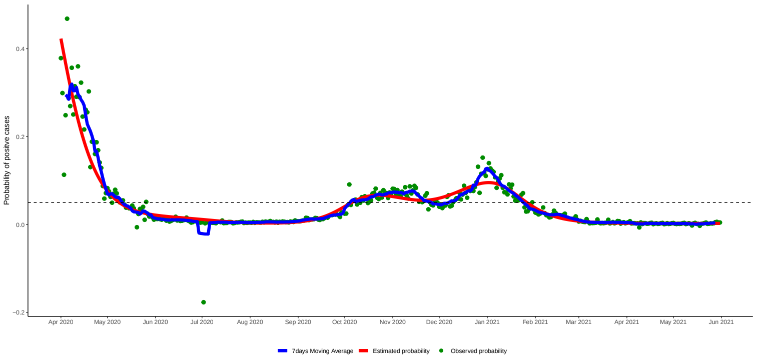

S11. Positive testing rate in the United Kingdom. Observed proportion of infection over time, estimated probability (red line) and a 7 days moving average (blue line).

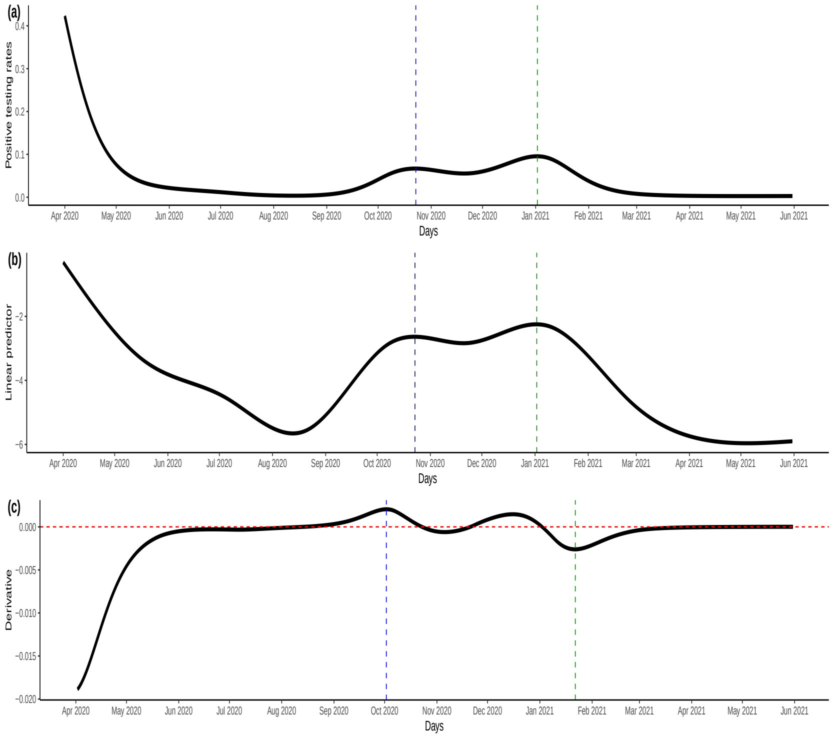

S12. United Kingdom result. Panel a: Estimated positive testing rate with 95% simultaneous confidence band. The blue and green vertical lines represent the peak (October 23rd, 2020 and January 2nd, 2021). Panel b: The linear predictor of the smoother with 95% simultaneous confidence band. The blue and green vertical lines represent the peak (October 23rd, 2020 and January 2nd, 2021). Panel c: The first order derivative of the estimated probability with 95% simultaneous confidence band between March 7th, 2020 and September 2nd, 2020. The blue and green vertical lines represent the peak (October 2nd, 2020 and January 22nd, 2021). while the red horizontal line represent the rate of change at 0.

References

E. Guidotti and D. Ardia. Covid-19 data hub. *Journal of Open Source Software*, 5(51):2376, 2020. doi: 10.21105/joss.02376.

R. Sengupta, R. Shome, P. Soumya, O. Owokotomo, and Z. Shkedy. *COVID-19 dashboard Multiple Countries*, 2021. URL https://rdsg.shinyapps.io/COVID19dashboardMC/.
